# Supplementary material for: Multi‐dataset identification of innovative feature genes and molecular mechanisms in keratoconus
Source: J Cell Mol Med. 2024 Sep 19;28(18):e70079. doi: 10.1111/jcmm.70079 (PMC11412914; doi:10.1111/jcmm.70079)
Supplement: Supplementary file 2 — Material S2. [file JCMM-28-e70079-s002.zip › 7.GSEA analysis/4.MYRF/KEGG_BASAL_CELL_CARCINOMA.html]

Details for gene set KEGG\_BASAL\_CELL\_CARCINOMA[GSEA]

|  || Dataset | MYRF.group.cls#h\_versus\_l |
| Phenotype | group.cls#h\_versus\_l |
| Upregulated in class | l |
| GeneSet | KEGG\_BASAL\_CELL\_CARCINOMA |
| Enrichment Score (ES) | -0.5935453 |
| Normalized Enrichment Score (NES) | -1.7286283 |
| Nominal p-value | 0.002096436 |
| FDR q-value | 0.18730386 |
| FWER p-Value | 0.329 |
Table: GSEA Results Summary

  

Fig 1: Enrichment plot: KEGG\_BASAL\_CELL\_CARCINOMA      
 Profile of the Running ES Score & Positions of GeneSet Members on the Rank Ordered List

  

| SYMBOL | TITLE | RANK IN GENE LIST | RANK METRIC SCORE | RUNNING ES | CORE ENRICHMENT || 1 | WNT10B | na | 5576 | 0.206 | -0.1403 | No |
| 2 | PTCH1 | na | 6108 | 0.189 | -0.1367 | No |
| 3 | APC2 | na | 8076 | 0.137 | -0.1798 | No |
| 4 | WNT16 | na | 8449 | 0.128 | -0.1777 | No |
| 5 | FZD1 | na | 9008 | 0.116 | -0.1823 | No |
| 6 | WNT5A | na | 9048 | 0.115 | -0.1719 | No |
| 7 | FZD3 | na | 10112 | 0.094 | -0.1932 | No |
| 8 | SUFU | na | 11793 | 0.066 | -0.2350 | No |
| 9 | FZD5 | na | 11924 | 0.065 | -0.2323 | No |
| 10 | WNT3 | na | 12453 | 0.057 | -0.2418 | No |
| 11 | TP53 | na | 12977 | 0.051 | -0.2518 | No |
| 12 | FZD4 | na | 13390 | 0.045 | -0.2592 | No |
| 13 | WNT2 | na | 13551 | 0.043 | -0.2595 | No |
| 14 | TCF7L2 | na | 15138 | 0.027 | -0.3026 | No |
| 15 | PTCH2 | na | 15175 | 0.026 | -0.3010 | No |
| 16 | FZD9 | na | 15491 | 0.023 | -0.3078 | No |
| 17 | CTNNB1 | na | 15600 | 0.022 | -0.3088 | No |
| 18 | HHIP | na | 15667 | 0.021 | -0.3086 | No |
| 19 | WNT8A | na | 17911 | 0.000 | -0.3732 | No |
| 20 | GSK3B | na | 19466 | -0.016 | -0.4165 | No |
| 21 | FZD6 | na | 19569 | -0.017 | -0.4177 | No |
| 22 | FZD8 | na | 20046 | -0.022 | -0.4292 | No |
| 23 | WNT4 | na | 20821 | -0.031 | -0.4484 | No |
| 24 | WNT3A | na | 20924 | -0.033 | -0.4481 | No |
| 25 | WNT9A | na | 21202 | -0.036 | -0.4525 | No |
| 26 | GLI3 | na | 21836 | -0.044 | -0.4664 | No |
| 27 | DVL1 | na | 22373 | -0.050 | -0.4768 | No |
| 28 | DVL3 | na | 23247 | -0.063 | -0.4957 | No |
| 29 | AXIN1 | na | 23328 | -0.065 | -0.4915 | No |
| 30 | SMO | na | 25095 | -0.095 | -0.5330 | No |
| 31 | WNT1 | na | 27000 | -0.135 | -0.5744 | No |
| 32 | TCF7L1 | na | 27666 | -0.152 | -0.5784 | Yes |
| 33 | STK36 | na | 27892 | -0.157 | -0.5691 | Yes |
| 34 | GLI2 | na | 27939 | -0.159 | -0.5546 | Yes |
| 35 | WNT10A | na | 28144 | -0.164 | -0.5440 | Yes |
| 36 | DVL2 | na | 28187 | -0.166 | -0.5287 | Yes |
| 37 | APC | na | 28648 | -0.180 | -0.5240 | Yes |
| 38 | WNT2B | na | 28930 | -0.189 | -0.5132 | Yes |
| 39 | WNT7B | na | 29404 | -0.203 | -0.5066 | Yes |
| 40 | TCF7 | na | 30107 | -0.227 | -0.5041 | Yes |
| 41 | FZD10 | na | 30271 | -0.233 | -0.4856 | Yes |
| 42 | WNT8B | na | 30486 | -0.242 | -0.4676 | Yes |
| 43 | WNT9B | na | 31651 | -0.298 | -0.4714 | Yes |
| 44 | WNT5B | na | 32169 | -0.328 | -0.4535 | Yes |
| 45 | WNT7A | na | 32275 | -0.335 | -0.4231 | Yes |
| 46 | FZD2 | na | 32361 | -0.340 | -0.3916 | Yes |
| 47 | AXIN2 | na | 32625 | -0.360 | -0.3632 | Yes |
| 48 | GLI1 | na | 32884 | -0.379 | -0.3328 | Yes |
| 49 | WNT6 | na | 33111 | -0.398 | -0.2996 | Yes |
| 50 | LEF1 | na | 33408 | -0.432 | -0.2650 | Yes |
| 51 | BMP4 | na | 33633 | -0.460 | -0.2255 | Yes |
| 52 | SHH | na | 33799 | -0.486 | -0.1817 | Yes |
| 53 | FZD7 | na | 34203 | -0.571 | -0.1362 | Yes |
| 54 | WNT11 | na | 34512 | -0.683 | -0.0768 | Yes |
| 55 | BMP2 | na | 34656 | -0.831 | 0.0021 | Yes |
Table: GSEA details [plain text format]

  

Fig 2: KEGG\_BASAL\_CELL\_CARCINOMA      
 Blue-Pink O' Gram in the Space of the Analyzed GeneSet

  

Fig 3: KEGG\_BASAL\_CELL\_CARCINOMA: Random ES distribution      
 Gene set null distribution of ES for **KEGG\_BASAL\_CELL\_CARCINOMA**
